# The prevalence, subtypes and associated factors of hyperuricemia in lupus nephritis patients at chronic kidney disease stages 1–3

**DOI:** 10.18632/oncotarget.19051

**Published:** 2017-07-06

**Authors:** Simeng Liu, Yijun Gong, Hong Ren, Wen Zhang, Xiaonong Chen, Tong Zhou, Xiao Li, Nan Chen

**Affiliations:** ^1^ Department of Nephrology, Ruijin Hospital, Shanghai Jiao Tong University School of Medicine, Shanghai, China; ^2^ HBI Solutions Inc, Palo Alto, CA, USA

**Keywords:** hyperuricemia, lupus nephritis, prevalence, subtypes, associated factors

## Abstract

There is a high prevalence of hyperuricemia (HUA) in the chronic kidney disease (CKD) population. However, there’s a dearth of research on HUA’s prevalence, subtypes, early detection, and treatment strategies of HUA in lupus nephritis (LN) patients. The aim of this study is to address these knowledge gaps. LN patients presenting to the Department of Nephrology at Shanghai Rui Jin Hospital from January 2011 to January 2016 were recruited. The effective sample size was derived using the power analysis. The demographic, clinical and laboratory characteristics of the LN patients with HUA were compared with those of patients without HUA. Two statistical models for analyzing HUA were built and compared using the receiver operating characteristic (ROC) curve analysis. The total prevalence of HUA in the cohort was 40.11%. The subtypes of HUA included urate underexcretion-type, overproduction-type and combined-type, which proportion being 67.7%, 9.7% and 22.6% respectively. The CKD stage was closely associated with the prevalence of HUA in patients with LN. The other significant associated factors were hypertension, triglycerides, serum creatinine, serum albumin, hemoglobin, parathyroid hormone, phosphorus, calcium, etc. The statistical algorithm successfully identified LN patients at risk of HUA. In conclusion, there was a high prevalence of HUA in LN patients at CKD stages 1–3, and renal underexcretion hyperuricemia was the most prevalent subtype. The occurrence of HUA in LN may be related to renal insufficiency, metabolic disorder and lupus itself. Early care coordination programs can employ risk models to improve HUA prevention and target interventions in LN patients.

## INTRODUCTION

Systemic lupus erythematosus (SLE) is a highly complex autoimmune disorder involving in multisystem injuries, which usually occurs in females of childbearing age [1]. Lupus nephritis (LN) accounts for significant morbidity and mortality in SLE patients [2]. Several studies have shown high metabolic syndrome (MS) prevalence in patients with SLE, varying from 18% to 45.2% [3–5], and the patients with SLE and MS usually had a significantly increased level of serum uric acid (SUA). However, few studies have been published focusing on the prevalence of hyperuricemia (HUA) in LN patients.

HUA is defined as a serum urate concentration greater than 416 μmol/l (7mg/dl) in men and postmenopausal women while greater than 357 μmol/l (6 mg/dl) in premenopausal women. It’s closely associated with a variety of diseases such as gout, hypertension, diabetes, obesity, coronary heart disease and kidney disease [6, 7]. It was reported that SUA level was independently associated with the progression of LN and prediction of future development of pulmonary hypertension in SLE patients [8, 9]. Urate lowering therapy (ULT) could dramatically modify the course of renal disease in HUA patients with chronic kidney disease (CKD) and the risk of renal disease progression can be reduced by 69.4% with SUA level < 420 μmol/l [10]. Therefore, greater emphasis needs to be placed on CKD patients with HUA, even during the early stages of the disease.

HUA is usually caused by an overproduction of urate and/or inefficient renal clearance of it. So far, no studies have ever assessed the subtypes of HUA in the LN patients. Fractional excretion of uric acid (FEua) is the ratio of renal urate clearance to creatinine clearance. Based on the results of FEua, HUA could be clinically classified into the urate ‘overproduction’ subtype (FEua > 12 %), the ‘underexcretion’ subtype (FEua < 7%), and the ‘combined’ subtype (7% ≤ FEua ≤ 12%) [11]. Besides, FEua has been supposed to identify individuals whose renal tubules, inherently, are more responsible for the inefficiency in clearing urate from the blood [12]. It is approximately independent of glomerular kidney function with the estimated glomerular filtration rates (eGFR) above 30 ml/min, and the FEua increases disproportionately with decreasing creatinine clearance while eGFR below 30 ml/min [13].

In light of the above we hypothesized that a CKD staging process is closely associated with the prevalence of HUA in patients with LN. This process is uric acid (UA) level driven resulting in the production of as yet unidentified associated factors in blood and urine measurements. The specific aims of this study were to examine the prevalence of HUA in patients with LN at CKD stages 1–3 and the differences between LN patients with and without HUA, to evaluate the excretion of UA in the LN patients with HUA, to identify the associated factors for HUA in the LN patients with early renal damage and to develop statistical models for analyzing HUA.

## RESULTS

### Characteristics of LN patients with or without HUA

A total of 177 LN patients were included into this study. The prevalence of HUA in the cohort was 40.11%. Demographic, clinical, laboratory and pathological characteristics of LN patients with or without HUA were shown in Table 1 and Table 2. As the two tables showed, there were more hypertension, a higher proportion of urine sediment, higher levels of SUA, blood urea nitrogen (BUN), serum creatinine (Scr), triglycerides, blood glucose, phosphorus, parathyroid hormone (PTH), 24 h urinary albumin, 24 h urinary α1-microglobulin, urine N-acetyl-β-D-glucosaminidase (NAG) activity, lower levels of eGFR, serum album, complement 3 (C3), 24 h urinary calcium, urinary volume and urinary pH in LN patients with HUA than those without HUA. However, there was no significant difference between the two groups in other laboratory parameters such as cholesterol, low density lipoprotein (LDL), immunoglobulin G/A/E/M (Ig G /A/E/M), complement 4 (C4) and etc. Renal biopsy was performed in 141 patients. In the group with HUA, the patient number of the pathological class I, II, III, IV, V, (II + V), (III + V), (IV + V) were 1, 0, 7, 19, 5, 0, 12, 9 respectively; In the group without HUA, the patient number of class I to (IV + V) were 1, 9, 10, 18, 19, 3, 20, 8 respectively. There was a statistical difference in the constituent ratio of pathological types between the two groups (*P* < 4.5%). There was more class IV in the group with HUA.

**Table 1:** The demographic, clinical and pathological characteristics of LN patients at CKD stages 1–3

Characteristics	Overall (*N* = 177)	HUA (*N* = 71)	Non-HUA (*N* = 106)	*P* value
Female (%)	149.0 (84.2)	56.0 (78.9)	93.0 (87.7)	0.117
Age (yr; median [range])	37.5 (26.0,49.0)	37.0 (26.0,49.0)	37.9 (26.0,50.0)	0.521
Course (mo; median [range])	50.6 (2.0,60.0)	53.6 (1.0,48.0)	48.6 (2.0,63.0)	0.690
Hypertension (%)	77.0 (43.5)	40.0 (56.3)	37.0 (34.9)	0.005
Hyperlipidemia (%)	71.0 (40.1)	30.0 (42.3)	41.0 (38.7)	0.635
Diabetes (%)	8.0 (4.5)	1.0 (1.4)	7.0 (6.6)	0.078
ACEI/ARB (%)	75.0 (42.4)	35.0 (49.3)	40.0 (38.1)	0.199
Body Mass Index (Kg/ m^2^ ; mean ± SD)	22.9 ± 3.8	23.4 ± 3.4	22.6 ± 4.0	0.071
CKD stages				< 0.001
Stage 1 (%)	91.0 (51.4)	23.0 (32.4)	68.0 (64.2)	
Stage 2 (%)	50.0 (28.2)	24.0 (33.8)	26.0 (24.5)	
Stage 3 (%)	36.0 (20.3)	24.0 (33.8)	12.0 (11.3)	
SLE-DAI (median [range])	10.0 (8.0,13.5)	12.0 (9.0,16.0)	10.0 (6.0,13.0)	0.307
Crescents (%)	69.0 (48.9%)	36.0 (67.9%)	33.0 (37.5%)	0.053
Global sclerosis (%)	88.0 (62.4%)	38.0 (71.7%)	50.0 (56.8%)	0.055
Mesangial proliferation [M-S (%)]	49.0 (34.8%)	20.0 (37.7%)	29.0 (33.0%)	0.345
Endothelial proliferation [M-S (%)]	47.0 (33.3%)	22.0 (41.5%)	25.0 (28.4%)	0.079
Leukocyte infiltration [M-S (%)]	26.0 (18.4%)	13.0 (24.5%)	13.0 (14.8%)	0.180
Tubular interstitial lesions [M-S (%)]	103.0 (73.0%)	41.0 (77.4%)	62.0 (70.5%)	0.244
Small vascular lesions (%)	63.0 (44.7%)	25.0 (47.2%)	38.0 (43.2%)	0.387

M-S = moderate to severe.

**Table 2:** The laboratory characteristics of LN patients at CKD stages 1–3

Variable	Overall (*N* = 177)	HUA (*N* = 71)	Non-HUA (*N* = 106)	*P* value
Blood glucose (mmol/l; mean ± SD)	4.4 ± 0.9	4.5 ± 0.7	4.3 ± 0.90	0.036
Hemoglobin (g/l; mean ± SD)	110.1 ± 20.4	106.2 ± 19.5	112.8 ± 20.6	0.04
Serum albumin (g/l; mean ± SD)	25.9 ± 8.6	24.2 ± 8.5	27.0 ± 8.5	0.045
Scr (μmol/l; median[range])	74.0 (60.0,99.0)	93.0 (71.0,125.0)	68.0 (53.8,80.0)	< 0.001
SUA (μmol/l; mean ± SD)	362.0 ± 114.9	473.9 ± 80.3	287.1 ± 60.9	< 0.001
Triglycerides (mmol/l; mean ± SD )	2.8 ± 1.7	3.1 ± 1.6	2.6 ± 1.7	0.006
Cholesterol (mmol/l; mean ± SD)	5.6 ± 2.6	5.7 ± 2.7	5.6 ± 2.5	0.973
Calcium (mmol/l; mean ± SD)	2.0 ± 0.2	2.0 ± 0.2	2.1 ± 0.2	0.032
Phosphorus (mmol/l; mean ± SD)	1.3 ± 0.3	1.4 ± 0.3	1.3 ± 0.3	0.003
25-OH-VitD (nmol/l; median[range])	23.2 (12.7,35.3)	22.5 (10.2,35.2)	23.6 (14.6,35.4)	0.510
PTH(pg/ml; median[range])	32.9 (21.2,53.8)	39.6 (23.4,77.1)	30.4 (19.8,46.5)	0.003
Anti-dsDNA(IU/ml; median[range])	310.5 (84.2,838.3)	426.3 (102.5,737.6)	273.0 (58.9,578.8)	< 0.001
C3(mg/dl; mean ± SD)	55.5 ± 28.4	48.4 ± 29.3	60.3 ± 27.0	< 0.001
Urine sediment [(RBC > 3/HP)%]	98.0 (55.4)	48.0 (67.6)	50.0 (47.2)	0.007
24 h urinary albumin (mg; median[range])	1522.0 (305.5,4417.5)	2676.0 (306.0,5341.0)	1233.0 (225.3,3099.5)	0.023
24 h urinary microalbumin (mg/24 h; median [range])	1160.0 (296.4,3181.0)	2137.5 (506.8,3627.0)	769.5 (189.0,2800.0)	0.298
24 h urinary -α1- microglobulin (mg/24h; median [range])	26.5 (12.4,55.4)	33.5 (16.3,70.9)	20.6 (11.7,41.3)	0.035
24 h urinary creatinine (mmol/24 h; median [range])	7.9 (6.2,10.2)	7.9 (5.8,11.3)	7.8 (6.4,9.8)	0.829
24 h urinary urea (mmol/24 h; mean±SD)	223.1 ± 75.1	219.5 ± 78.9	225.7 ± 72.7	0.350
24 h urinary uric acid (mmol/24 h; median [range])	2.4 (1.9,3.0)	2.3 (1.8,2.7)	2.5 (1.9,3.1)	0.313
24 h urinary calcium (mmol/24 h; median [range])	1.0 (0.5,2.3)	0.6 (0.4, 1.0)	1.5 (0.8, 2.9)	< 0.001
24 h urinary phosphorus (mmol/24 h; mean ± SD)	10.5 ± 4.7	10.4 ± 4.8	10.7 ± 4.6	0.615
RBP (mg/l; median [range])	1.9 (0.4,4.6)	1.8 (0.5,4.5)	2.0 (0.3,4.67)	0.785
NAG activity (U/L; median [range])	15.1 (6.8,26.2)	21.4 (9.8,38.0)	13.2 (5.5,20.8)	< 0.001
U-ACR (mg/mmol; median [range])	142.1 (45.9,405.9)	215.9 (73.6,478.7)	110.8 (38.5,621.3)	0.361
Urinary pH (median [range])	6.5 (5.0,7.0)	6.0 (5.0,6.8)	6.5 (6.0,7.0)	0.018
Urinary volume (mean ± SD)	1.3 ± 0.5	1.2 ± 0.5	1.4 ± 0.5	0.009
eGFR (ml/min/1.73 m^2^; mean ± SD)	85.5 ± 30.8	75.7 ± 25.0	98.7 ± 28.5	< 0.001
FEua (%; median [range])	4.9 (3.5,9.2)	4.6 (3.4,9.2)	4.9 (9.2,3.5)	0.175

### Associated factors for HUA in LN patients

**Figure 1:**
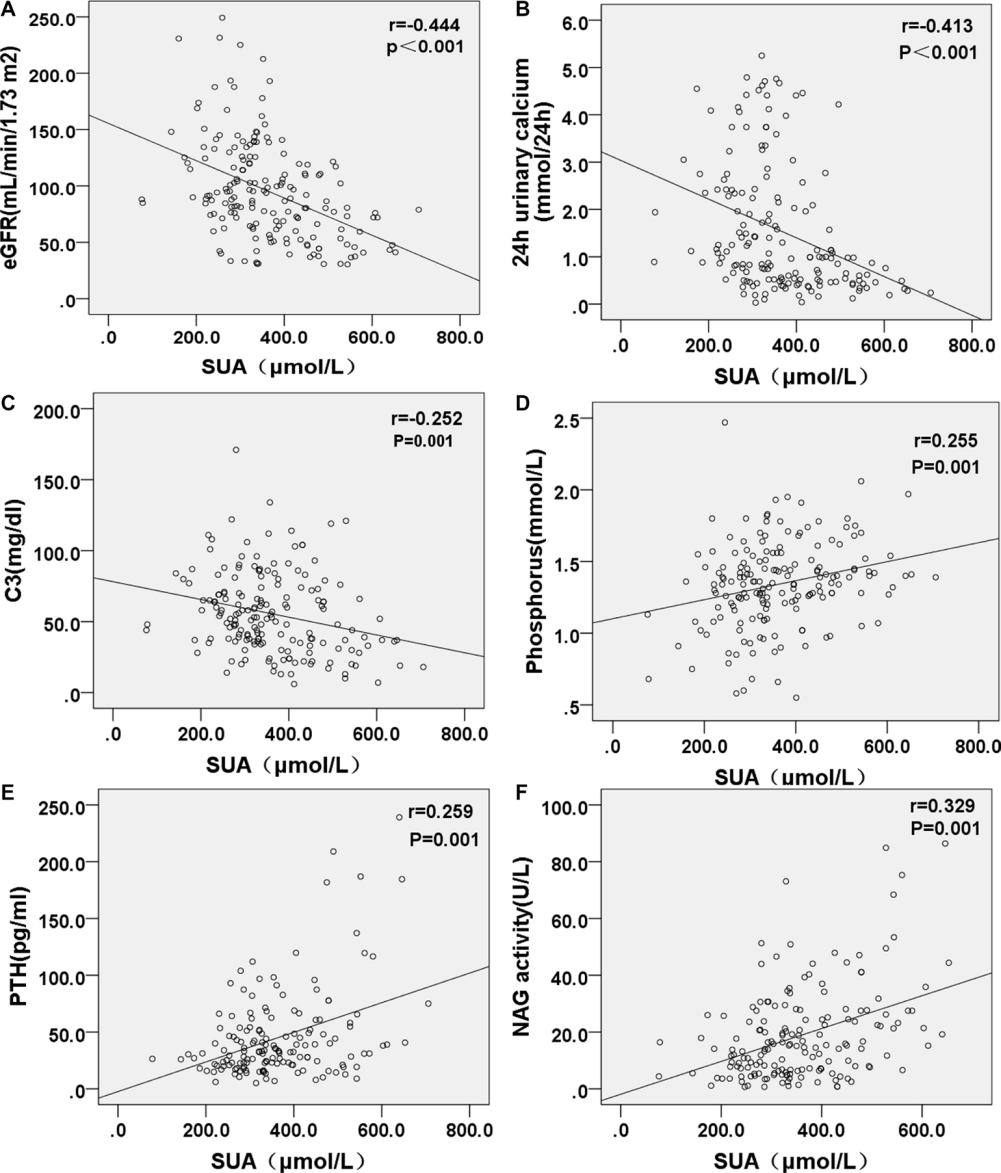
Correlations of SUA with eGFR, 24 h urinary calcium, C3, phosphorus, PTH and NAG Estimated glomerular filtration rate (eGFR), 24 h urinary calcium and serum C3 level were negatively correlated with serum UA level, while serum phosphorus, PTH levels and urine NAG activity being positively correlated with serum UA level.

The correlations between the variables and SUA were presented in Table 2, Table 3 and Figure 1. Univariate logistic regression was used to analyze the HUA risk. CKD stage was closely associated with the prevalence of HUA in patients with LN. The other significant associated factors were hypertension, eGFR, blood glucose, triglycerides, BUN, Scr, serum albumin, hemoglobin, PTH, phosphorus, calcium, C3, urine sediment, urine NAG activity, 24h urinary calcium, 24 h urinary albumin, 24 h urinary α1-microglobulin, urinary pH and urinary volume. *P* values, Odds ratios (OR) and 95% CI for each associated variable were listed in Table 3.

**Table 3:** Significant associated factors for HUA and their correlations with SUA level

Variable	Associations with HUA				Correlations with SUA	
	OR	95% CI		*P* value	*r* value	*P* value
Hypertension	2.41	1.3	4.46	0.005		
eGFR (ml/min/1.73 m^2^)	0.21	0.1	0.41	< 0.001	−0.444	< 0.001
CKD stage	2.47	1.64	3.72	< 0.001		
Blood glucose (mmol/l)	1.95	1.02	3.72	0.036	0.217	0.004
Hemoglobin (g/l)	0.53	0.29	0.98	0.04	−0.197	0.009
Scr (μmol/l)	7.48	3.48	16.09	< 0.001	0.440	< 0.001
BUN (mmol/l)	11.3	4.75	26.89	< 0.001	0.534	< 0.001
Triglycerides (mmol/l)	2.37	1.27	4.45	0.006	0.223	0.003
Calcium (mmol/l)	0.51	0.27	0.96	0.032	−0.205	0.006
Phosphorus (mmol/l)	2.6	1.32	5.12	0.003	0.255	0.001
PTH (pg/ml)	2.57	1.34	4.91	0.003	0.259	0.001
C3 (mg/dl)	0.32	0.16	0.61	< 0.001	−0.252	0.001
Urine sediment [(RBC > 3/HP)%]	3.4	1.38	8.4	0.007		
NAG activity (U/l)	2.93	1.5	5.73	< 0.001	0.329	< 0.001
24 h urinary albumin (mg)	2.11	1.07	4.15	0.023	0.231	0.002
24 h urinary -α1- microglobulin (mg/24 h)	1.92	1.04	3.56	0.035	0.257	0.002
24 h urinary calcium (mmol/24 h)	0.13	0.06	0.29	< 0.001	−0.413	< 0.001
Urinary pH	0.48	0.26	0.89	0.018	−0.222	0.003
Urinary volume	0.44	0.24	0.83	0.009	−0.216	0.004

The stepwise multivariate logistic regression was performed to select significant associated factors based on the AIC criteria. *P*-value < 4.5% was declared statistically significant with FDR controlled at 5% level.

### Two statistical models detecting LN patients at risk of HUA

**Figure 2:**
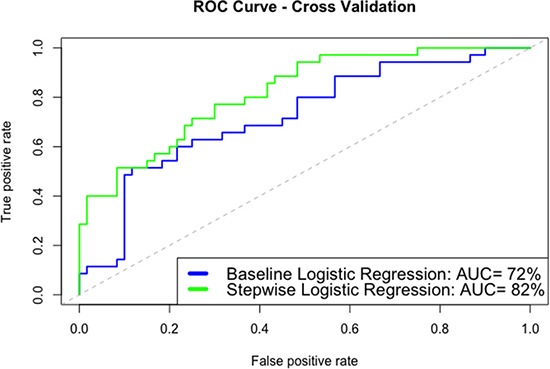
ROC analysis of the baseline model and the stepwise logistic regression model The stepwise logistic regression model (AUC = 0.82) performed better than the baseline logistic model (AUC = 0.72) by cross validation.

Receiver operating characteristic (ROC) curves (Figure 2) were generated to evaluate the model performances. The two statistical models performed well on detecting LN patients at risk of HUA. The stepwise logistic regression model (AUC = 0.82) performed better than the baseline logistic model (AUC = 0.72) by cross validation. The equation for the selected stepwise logistic regression model is given below.

$\log\left(\frac{P}{1-P}\right)=-0.5+2.5\text{ BUN}+0.7\text{ PTH}-0.9(24\;h\text{ urinary calcium})+0.9\text{ Triglycerides}-0.7\;C3+0.6(24\;h\text{ urinary albumin})-0.8(24\;h\text{ urinary al- microglobulin})$, where *P* refers to the probability of HUA.

### The subtypes of HUA in patients with LN

**Figure 3:**
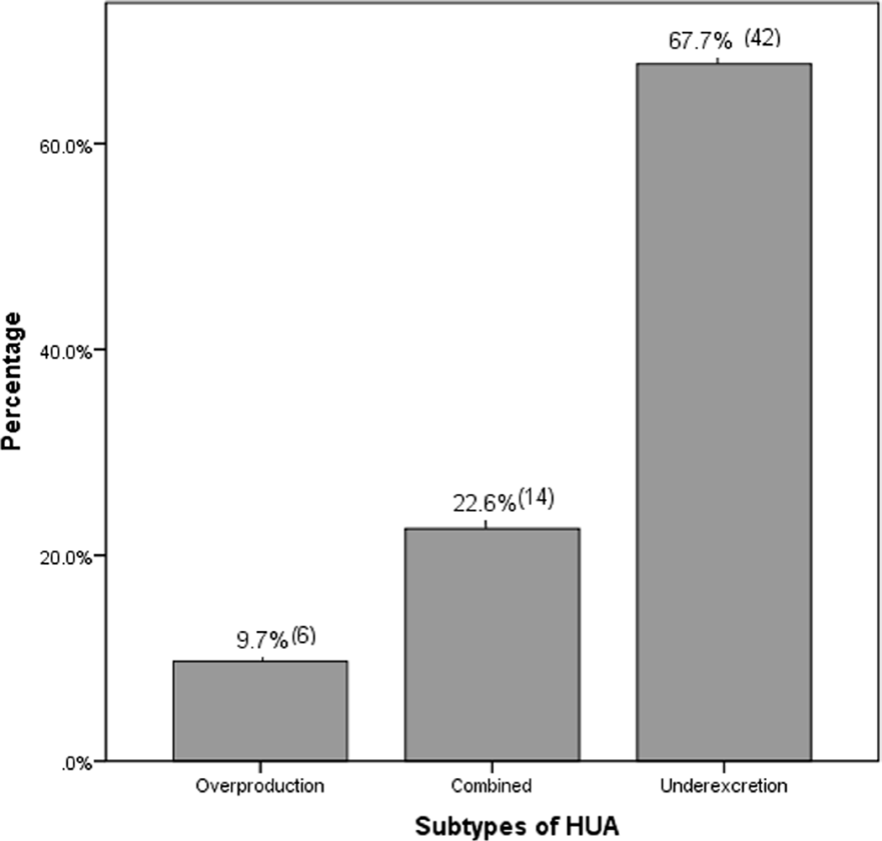
The subtypes of HUA in patients with LN at CKD stages 1–3 The proportion (%) and the numbers of the patients in different subtypes of HUA were described. Urate underexcretion was the major subtype of HUA in LN patients at CKD stages 1–3 (*P* < 0.001).

The different subtypes of HUA in LN patients at CKD stages 1–3 were summarized in Figure 3. Renal urate underexcretion-type, urate overproduction-type and the combined-type were 67.7%, 9.7% and 22.6% respectively. Urate underexcretion was the main cause of HUA in this population (*P* < 0.001).

## DISCUSSION

The prevalence of HUA increased with the CKD staging process. Compared with the general CKD population, the prevalence of HUA was much higher in LN patients. Data from a community-based population study including 187 914 participants showed the prevalence of HUA at CKD stages 1–3 was 23.3% [14], while it being 40.11% in LN patients at CKD stages 1–3. The actual figures may be even larger because those patients using medications affecting SUA levels were excluded in this study. Sabio *et al* reported that the prevalence of HUA was just 15% in a cohort of 102 SLE patients mainly attributing to mild SLE (only 37% patients with LN at CKD stages 1–2) [15].

This study has several strengths that merit consideration. The inclusion of demographic, clinical and pathological features were broad and diverse. LN patients with HUA usually have more serious clinical manifestations than patients without HUA. This study proved that the triglyceride was one of the significant associated factors of HUA in patients with LN at CKD stages 1–3. UA was recognized as a by-product of MS that is a cluster of cardiovascular risk factors such as hypertension, dysglycemia, dyslipidemia, etc [4]. A recent study suggested that the SUA was also a pathogenic factor for MS [16]. Glut 9-deficient mice were used to develop early-onset spontaneous HUA model, that dyslipidemia, hyperinsulinemia and hepatic fat deposition began to occur in 6–8 weeks, and allopurinol could reverse the hypertension and hypercholesterolemia [16]. We also found there were much more patients suffering hypertension in HUA group. Animal models and several small trials showed the reduction of SUA could reduce blood pressure [17, 18]. In fact, HUA was demonstrated to cause hypertension via a chain of events including a reduction in nitric oxide synthase, activation of the renin–angiotensin system (RASS) and reduction of renal perfusion, leading to increased systemic vascular resistance, resulting in a late sodium-sensitive hypertension. Importantly, each of these effects was ameliorated by UA lowering therapy [17–20].

The association between SUA level and bone mineral metabolism was explored in this study. Most of the patients had a low level of 25-OH-Vitamin D (25-OH-VitD). The patients with HUA had a lower level of serum calcium, a higher level of serum phosphorus and PTH than those without HUA. Avoiding direct sunlight in the daily life might play a role in the deficiency of serum 25-OH-VitD and calcium in LN patients. Considering the widely insufficiencies of 25-OH-VitD in the patients of this study, the impact of vitamin D or its metabolites on HUA was needed to clarify in future prospective studies. It was reported that serum PTH levels were independently associated with SUA levels and the frequency of HUA in the general population [21]. PTH might have negative effects on the net proximal tubular urate reabsorption, given that proximal tubular salt (Na^+^-Cl^−^) and urate transport are regulated in parallel [22]. Interestingly, we found there was a significant and inverse relationship between 24 h urinary calcium and SUA, possibly due to co-localization of anion transporters and uric acid transporters (URAT1) [22, 23].

Urate is filtered via the glomeruli, then reabsorbed and also secreted by the renal tubules [23]. Therefore, a diminished GFR, increased tubular resorption, impaired tubular secretion, or a combination of both disrupts SUA homeostasis and eventually aggravates HUA [24]. This study proved the high level of SUA was correlated with the impaired glomerular and renal tubular function. HUA was not only a consequence of renal insufficiency, but also a cause of renal injuries. Traditionally, it has been postulated that SUA causes kidney disease by intra-luminal crystal depositing in the collecting duct of the nephron. It has been reported experimentally that UA may contribute to renal arteriolopathy and tubulointerstitial fibrosis mainly by inducing inflammation, endothelial dysfunction, oxidative stress, and activation of RASS [19]. It has also been shown that SUA can activate the cytoplasmic phospholipase A2 and the inflammatory transcription factor nuclear factor κ B (NF-κB), resulting in the inhibition of proximal tubular cellular proliferation *in vitro* [23]. ULT was reported to reduce tubulointerstitial fibrosis both in the 5/6th nephrectomy model and in diabetic nephropathy [25]. The obvious differences were not found in renal pathological changes between the two groups, and the higher proportion of pathological class IV may account for disease activity in the HUA group.

This study found that SUA level was negatively correlated with C3, which was consistent with the previous study [8, 26]. The explanation might be that elevated UA in LN can activate C3 through classical and alternative pathways, as described in previous studies *in vitro* [27]. The deposition of complement activation products, in turn, aggravated the renal tissue injury and development of LN. A previous study provided dramatic new insights into the role of UA [28], showing that UA is a principal endogenous danger signal released from injured cells that activates the immune system.

24 h urinary uric acid, uric acid clearance (Cur) as well as FEua can reflect the excretion of UA. 24 h urinary uric acid could be easily affected by the intake of high-purine diet, drinking of water, urinary volume, renal function and the level of SUA. On the contrary, FEua eliminates these confounding factors above [13]. The renal clearance of urate decreases with decreasing creatinine clearance down to about 30 ml/min, FEua remains quite stable and increases only marginally [13]. Hence FEua is a more accurate descriptor of the ratio of clearance of urate to creatinine than the alternatives [29, 30]. Although FEua alone was not predictive of HUA, a low FEua may be a risk factor for subsequent development of HUA [30]. In this study, urate underexcretion was the dominant subtype of HUA in LN patients at CKD stages 1–3, accounting for 67.2%. Pinpointing the causes of HUA would help to provide a more accurate and effective therapeutic strategy.

In addition, the statistical algorithm we developed successfully identified LN patients at risk of HUA. A few variables stood out as being important factors for HUA detection. With only several major variables, the enhanced risk model is capable of estimating the probability of HUA more accurately. Compared with the baseline model, the stepwise logistic regression model enhanced the AUC from 0.72 to 0.82. By using this statistical algorithm, whether a LN patient is at risk of HUA could be reliably and easily assessed.

The results of this study should be interpreted with respect to several limitations. Firstly, this was a single-center study, and our findings may be specific to Chinese patients, which might limit the generalizability to a wider population. Secondly, the intake of some food that can influence SUA levels was not taken into account. Thirdly, the models that we used relied on cross-sectional information found in blood samples and urine specimens. It is expected that the information held within the longitudinal data would improve the detection and prediction of HUA. Hence, further studies on prospective cohorts would be necessary to achieve more accurate results.

In conclusion, this study was first to consider the prevalence, subtypes and associated factors of HUA in LN patients at CKD stages 1–3. We found there was a high prevalence of HUA and underexcretion of UA was the dominant HUA subtype in LN patients at CKD stages 1–3. The occurrence of HUA in LN may be related to renal insufficiency, metabolic disorder and lupus itself. This study also pioneered to detect HUA in LN patients at CKD stages 1–3 with statistical modeling methods. Laboratory values and comorbidities were found to be important for detecting HUA-risk patients. Early care coordination programs can employ risk models to target interventions and improve HUA prevention in LN patients.

## MATERIALS AND METHODS

### Cohort construction

From January 2011 to January 2016, a total of 177 patients with LN in Department of Nephrology, Shanghai Rui Jin Hospital were enrolled when meeting the following inclusion criteria: (1) met the diagnosis criteria of SLE defined by the American Rheumatology Association in 1997 and diagnosed as LN according to clinical manifestation, laboratory findings or renal histopathological change. (2) satisfied the criteria for CKD stages 1–3 according to the Kidney Disease Improving Global Outcomes (KDIGO) clinical practice guidelines. All the patients were admitted after providing informed consent. Reasons for exclusion were drug-induced SLE, post-renal transplantation, merging malignant tumor and usage of medications affecting SUA levels (diuretics, allopurinol, febuxostat, benzbromarone, glucocorticoid and immunosuppressants, etc) before taking the blood and urine samples. The study was approved by the Ethics Committee of Shanghai Rui Jin Hospital.

### Clinical data collection

All the patients enrolled in the study underwent detailed clinical and laboratory assessments. Blood samples and urine specimens were obtained for detection of blood glucose, lipids, bone mineral metabolism indexes, Scr, BUN, SUA, urinary albumin creatinine ratio (U-ACR), 24 h urinary creatinine, 24h urinary uric acid, urinary retinol binding protein (RBP), urine NAG activity, anti-double-stranded DNA (anti-dsDNA) autoantibody, Ig G/A/E/M and C3, etc. Other information such as age, gender, height, weight, disease course, clinical manifestations, blood pressure and medications were also recorded.

### Pathologic data collection

In accordance with International Society of Nephrology/Renal Pathology Society (ISN/RPS) 2003 classification of LN, it can be divided into class I (minimal mesangial lupus nephritis), class II (mesangial proliferative lupus nephritis), class III (focal lupus nephritis), class IV (diffuse lupus nephritis), class V (membranous lupus nephritis) and class VI (advanced sclerosis lupus ephritis) [31]. All pathological changes were assessed by two professional pathology experts and could be separated into "mild-moderate-severe". The grading of renal lesions was done according to the following criteria: (1) mesangial hypercellularity with a score of 0, 1, or 2, respectively, for the different number of mesangial cells per area, (2) endocapillary proliferation scored as absent, involving < 50%, or involving > 50% of glomeruli, (3) crescent, assessed by the percentage of glomeruli affected, (4) sclerosis, assessed by the percentage of glomeruli affected, (5) leukocyte infiltration, for the number of polymorphonuclears and mononuclears detected, (6) the tubular atrophy and interstitial fibrosis in cortex were also estimated in percentages and were graded as 0, 1, 2 ,3(when these were absent, < 25%, 25% to 50% and ≥ 50%) and (7) hyaline arteriolosclerosis was recorded for arteriolar hyaline changes, with or without smooth muscle hyperplasia.

### Definitions

The systemic lupus erythematosus disease activity index (SLEDAI) score [32] was determined on the day of blood draw. Estimated GFR (eGFR) was calculated according to the chronic kidney disease epidemiology collaboration (CKD-EPI) equation taking gender, age and Scr into account [33]. Based on the KDIGO clinical practice guidelines, CKD 1 was defined as eGFR ≥ 90 ml/min/1.73 m^2^, CKD 2 was defined as eGFR 60–89 ml/min^/^1.73 m^2^ and CKD 3 was defined as eGFR 30–59 ml/min^/^1.73 m^2^. FEua was calculated as the ratio of urate clearance to creatinine clearance.

FEua = (Uua × V/ Sua)/(Ucr × V/Scr)
The volume (V) terms cancel out, leaving the simplified following formula.
FEua = (Uua × Scr)/(Sua × Ucr)
Where U and S represent urinary and serum concentrations, ua is urate, and cr is creatinine.

### Statistical Methods

### Study Setting

The study comprised of discovery and validation phases. Those with HUA diagnosis were compared with those without. To facilitate comparisons across follow-up studies, Rice and Harris [34] showed that area under curve (AUC) is the preferred measure of predictive or diagnostic accuracy in forensic psychology or psychiatry. In this study, we used AUC to characterize effect sizes. The normal approximation method was used in calculating the required sample size for the comparison of AUC with a null hypothesis value. Further, since there’s no data available, the variance of AUC was estimated based on the binormal assumption [35]. Thus, the power calculation [35] suggested that with a sample size *n* = 64 per group, a power of > 80% can be achieved at the 0.5% false positive level. To evaluate the predictive power of our risk model against a random classifier, the sample size calculation was based on the assumptions that our risk model should achieve a predictive ability in terms of AUC of 0.75 in distinguishing HUA from non-HUA. An estimated 10% loss to follow up results in a minimum 71 patients per group.

### Data Cleaning

Summary statistics were estimated for all variables and patients, and any missingness was identified. 14 variables (< 19%) had missing data on > 15% patients. This was primarily due to patients having moved during the study to other practices. After removing these 14 variables from the data, a small proportion of patients (< 5%) had missing data on primary measurements. Based on the assumption that the data is missing completely at random (MCAR), median imputation was used for all missing data.

### Statistical analysis

The demographic, clinical and pathological characteristics were expressed as mean ± SD for normally distributed data, median with interquartile ranges for non-normally distributed data and frequency (%) for categorical data. Correlation analysis between the variables and SUA was performed using Pearson’s correlation coefficient or Spearman’s rank correlation coefficient. Further, a logistic regression was applied to assess the effect of each variable on the prevalence of HUA in LN. The use of false discovery rate (FDR) control in the context of multiple testing provides a solid basis for drawing conclusions about statistical significance [36]. The bootstrap resampling method was used to estimate FDRs at sequential *P*-values. *P*-value < 4.5% was thus declared significant with FDR controlled at 5% level. Variables with *P*-value < 4.5% were considered statistically significant. The baseline multivariate logistic model was built using the selected variables. The stepwise multivariate logistic regression was performed and several variables were removed based on the akaike information criterion (AIC) criteria. These two models were compared by the area under the receiver operating characteristic (ROC) curve metric using cross validation. Statistical analysis was performed by using SPSS version 19.0 and RStudio version 0.99.893.

## Abbreviations

HUA: hyperuricemia
CKD: chronic kidney disease
LN: lupus nephritis
ROC: receiver operating characteristic
SLE: systemic lupus erythematosus
MS: metabolic syndrome
SUA: serum uric acid
ULT: urate lowering therapy
FEua: fractional excretion of uric acid
eGFR: estimated glomerular filtration rate
UA: uric acid
SLEDAI: systemic lupus erythematosus disease activity index
CKD-EPI: chronic kidney disease epidemiology collaboration equation
KDIGO: Kidney Disease Improving Global Outcomes
Scr: serum creatinine
BUN: blood urea nitrogen
U-ACR: urinary albumin creatinine ratio
RBP: retinol binding protein
PTH: parathyroid hormone
C3: complement3
C4: complement4
NAG: N-acetyl-β-D-glucosaminidase
RASS: renin–angiotensin system
ISN/RPS: International Society of Nephrology / Renal Pathology Society
AUC: Area Under Curve
FDR: false discovery rate
MCAR: missing completely at random
AIC: akaike information criterion
OR: odds ratio
95% CI: 95% confidence interval
URAT1: uric acid transporters1
NF-κ B: nuclear factor κ B
Cur: clearance rate
